# Strengthening and expanding the capacity of health worker education in Zambia

**DOI:** 10.11604/pamj.2017.27.92.6860

**Published:** 2017-06-07

**Authors:** Charles Michelo, Joseph Mumba Zulu, Moses Simuyemba, Benjamin Andrews, Max Katubulushi, Benjamin Chi, Evariste Njelesani, Bellington Vwalika, Kasonde Bowa, Margaret Maimbolwa, James Chipeta, Fastone Goma, Selestine Nzala, Sekelani Banda, John Mudenda, Yusuf Ahmed, Lotti Hachambwa, Craig Wilson, Sten Vermund, Yakub Mulla

**Affiliations:** 1 University of Zambia, School of Medicine, Department of Public Health, Lusaka, Zambia; 2 Vanderbilt University, USA; 3 University of North Carolina at Chapel Hill, USA; 4 Lusaka Apex Medical School, Zambia; 5 University Teaching Hospital, Ministry of Health, Zambia; 6 Copperbelt University, School of Medicine, Zambia; 7 Cavendish University School of Medicine; 8 University of Alabama, USA

**Keywords:** Training, health care worker education, medical education partnership initiative

## Abstract

**Introduction:**

Zambia is facing a chronic shortage of health care workers. The paper aimed at understanding how the Medical Education Partnership Initiative (MEPI) program facilitated strengthening and expanding of the national capacity and quality of medical education as well as processes for retaining faculty in Zambia.

**Methods:**

Data generated through documentary review, key informant interviews and observations were analyzed using a thematic approach.

**Results:**

The MEPI program triggered the development of new postgraduate programs thereby increasing student enrollment. This was achieved by leveraging of existing and new partnerships with other universities and differentiating the old Master in Public Health into specialized curriculum. Furthermore, the MEPI program improved the capacity and quality of training by facilitating installation and integration of new technology such as the eGranary digital library, E-learning methods and clinical skills laboratory into the Schools. This technology enabled easy access to relevant data or information, quicker turn around of experiments and enhanced data recording, display and analysis features for experiments. The program also facilitated transforming of the academic environment into a more conducive work place through strengthening the Staff Development program and support towards research activities. These activities stimulated work motivation and interest in research by faculty. Meanwhile, these processes were inhibited by the inability to upload all courses on to Moodle as well as inadequate operating procedures and feedback mechanisms for the Moodle.

**Conclusion:**

Expansion and improvement in training processes for health care workers requires targeted investment within medical institutions and strengthening local and international partnerships.

## Introduction

The limited number and mix of human resources for health (HRH) has contributed to poor health systems performance in many low and middle income countries (LMICs) [1–3]. Of the 57 countries that face critical health worker shortages, 63% are in the Africa region [4]. In sub-Saharan Africa, there are approximately 18 doctors and 119 nurses per 100,000 population, compared to analogous corresponding figures of 280 and 980, respectively, in the U.S.A [1, 2]. Zambia is one of the countries facing the HRH crisis. The country has about less than half the HRH that it needs and fewer than 646 doctors and 6,096 nurses for a population of about 14 million [5, 6]. This would equate to about 5.4 doctors and 51 nurses per 100,000. This crisis has been exacerbated by the stress brought about by the double burden of disease, (communicable and non communicable) and in particular health burdens of HIV infection, which have intensified health system demands on the already overstretched infrastructure [7–9] . This crisis is as a result of limited numbers of graduating health professionals, “brain drain” of doctors to non-clinical positions and/or jobs outside of the country [10, 11], and reallocation of current staff to well-funded initiatives for specific diseases, including HIV [12].

Key strategies for addressing the HRH crisis as inscribed in the Zambia National Health Strategic Plan (2011-2015) include scaling up the recruitment as well as improving distribution and retention. Improving the capacity of training and education institutions is vital in increasing HRH [13]. As a way of contributing towards addressing the HRH need, the U.S. President's Emergency Plan for AIDS Relief in partnership with the Office of the U.S. Global AIDS Coordinator and the National Institutes of Health and the Health Resources and Services Administration announced the Medical Education Partnership Initiative (MEPI).The overarching mission of MEPI is to increase the quantity, quality and retention of medical school graduates with specific focus on addressing the health needs of national populations, through direct funding to African medical schools. The University of Zambia, School of Medicine (UNZASOM) was one of 13 medical schools in the 12 African countries that was competitively awarded a MEPI grant in 2010. The UNZA program is focused on selected high-priority training programs, with the goal of (1) improving the capacity of training for health care workers equipped to address the broad needs of the existing health system, (2) improve the overall quality of health care worker training, with a particular focus on the enhancement of training resources, and (3) enhancing the academic environment to better retain faculty. Implementation of the MEPI program is being led by UNZA and in partnership with three other Zambian medical schools: Copperbelt University (CBU), Lusaka Apex Medical University (LAMU), and Cavendish University (CU). This paper aimed at answering the following research question: how did the MEPI program facilitate strengthening and expanding of the national capacity and quality of medical education as well as processes for retaining faculty in Zambia.

## Methods

**The study site:** The study was conducted at UNZA, CBU, LAMU, CU, the Universities that are implementing the MEPI program. UNZA, LAMU and CU are located in Lusaka city (the capital city of Zambia) while CBU is located in Kitwe district in the Copperbelt province.

**The study design:** A qualitative case study design was used. The case study design is an empirical study design that investigates contemporary phenomena within a real-life context, where the boundaries between phenomena and context are not clearly evident and in which multiple sources of evidence are used [6]. The case study design was considered as appropriate for this study because the MEPI program was developed within a complex context, which involved social interactions, and which was dependent on multiple individuals as well as Universities. This case study design focused on the MEPI sties and it analysed the process of strengthening and expanding the national capacity and quality for health care worker education in Zambia before and after the implementation of the MEPI program.

**Data collection:** The data reported in this paper is from October 2010, the time implementation of the MEPI program started, to June 2013. Data were collected through review of documents, interviews with program staff and observations. The first (CM), second (JMZ) and third (MS) authors reviewed the documents, conducted the interviews and observations.

**Documentary review :** The program documents primarily written as part of the MEPI program design and/or implementation process were reviewed. Documents included program implementation progress reports for the period of two and half years, program monitoring and evaluation frameworks, site visit and coordinating program reports, and MEPI annual survey documents, standard operation procedures for the skills laboratory, Moodle platform and course evaluation or assessment reports.

**Key informant interviews:** Interviews were conducted with program managers at the Universities, coordinators of the new programs namely the skills laboratory, medical education (eLearning), the new master programs and the monitoring and evaluation manager. The interviews were recorded and later transcribed.

**Observations:** Observations aimed at documenting visible MEPI implementation-related activities such as infrastructure, asset registers and classes for the new master programs were conducted. These observations were made to validate information from the review of documents and key informant interviews.

**Data analysis:** Data analysis followed thematic analysis which is a method for identifying, analysing and reporting patterns (themes) within data. It organizes and describes data set in detail and goes further to interpret various aspects of the research topic [14]. The first step in analysing data was the development of codes. The first, second and third authors developed initial codes after reviewing the data from the documentary review and interviews several times to develop a sense of the whole dataset. Codes were then grouped into categories - groups of content that share a commonality - and these were then developed into broader themes as reflected in Table 1. The coding process was done with the help of NVIVO version 7. This involved interpreting the categories for their underlying meaning, and grouping categories according to patterns [15] (Table 1). Data gathered from the review of documents and interviews were then triangulated with information gathered through observations. Triangulation involved assessing the consistency and potential variations of findings by comparing data patterns across the material generated by different methods [15].

**Table 1:** Selected categories and themes

Categories	Themes
Developing new programs	Training more health care workers
**Leveraging existing opportunities**	
Enhancing Staff Development Fellowship	
Integration of technology for medical education	Improving the quality of medical training
Strengthening academic environment	Processes for retaining faculty
**Doctoral training support**	
Post-doctoral training support	
Increased workload among faculty members	
Not all courses uploaded onto Moodle	Implementation limitations
Limited operating procedures	
Limited feedback mechanisms	

**Ethics:** Ethical clearance to conduct the study was obtained from the University of Zambia Biomedical Research Ethics Committee (IRB 0001131 of IORG 0000774, reference number 005-19-14). Verbal consent was sought from all study informants. Further, detailed explanation of the research objectives was done to each study participant. Confidentiality during and after study was assured to the study participants through withholding informants' personal details.

## Results

In this section, we present the processes and systems for enhancing the capacity and quality of health care worker training and retention of faculty in Zambia which emerged following the implementation of the MEPI program. The findings have been organized around three broad themes namely: training more health care workers, improving the quality of medical training, and processes for retaining faculty.

**Training more health care workers:** Implementation of the MEPI program resulted into improved systems and increased opportunities for training health care workers in Zambia. Below the strategies that facilitated enhancement of training processes are outlined.

**Developing new programs:** Before the implementation of the MEPI program, the SOM had very few new postgraduate programs. However, following the implementation of MEPI, 14 new postgraduate programs were developed. The development of the new programs would potentially help address the huge HRH gap by increasing both numbers and skills of health care workers trained. The programs adopted a multi-disciplinary approach to health service provision by emphasizing both clinical as well as basic science courses. Leveraging on existing opportunities was the main strategy which facilitated the increase in master programs.

**Leveraging existing opportunities:** One opportunity which was leveraged on when developing the master programs was pre-existing partnerships that had been developed for medical education capacity building. For example Vanderbilt University, through the AIDS International Training and Research Program partnered with UNZA beginning in 2008 to develop new curricula for the M.Med clinical residency degrees and to strengthen the research training and HIV-related research projects. Similarly, the University of Maryland and the Tropical Health and Education Trust helped establish new postgraduate programs, namely Masters of Science in HIV Medicine as well as the three Masters of Medicine in Pathology, Psychiatry, and Anesthesiology. Other newly developed programs included six Masters of Science (Physiology, Biochemistry, Anatomy, Haematology, Clinical Pathology, Clinical Pathology), five for Masters of Medicine (Anesthesia, Pathology, Psychiatry, Ophthalmology, Infectious Disease), as well as dedicated Masters programmes for Clinical Pharmacology, Physiotherapy, Biomedical Science, and Public Health. These programs increased enrollment rates at postgraduate level. Following the implementation of MEPI in 2013 postgraduate enrolment increased by 241 students. Whereas another 250 students were enrolled in the new distance learning degree program for nurses. Another form of leveraging on existing opportunities took the form of differentiating the old Master in Public Health into specialized curriculum. MEPI has also helped enhance the training processes through facilitating the revision of the existing Masters in Public Health into specialized tracks. The program was reviewed and differentiated into five specialized curricula namely Population Studies, Environmental Health, Epidemiology/Biostatistics, Health Promotion as well as Health Policy and Management. This helped to increase enrolment more than threefold from 15 candidates per year to 50 (10 per specialization tract).

**Enhancing staff development fellowship:** Staff shortage at the UNZA SOM has been a long standing challenge. The following percentages show some of vacant positions at the UNZA: 87% for professor, 94% for associate professor, 82% for senior lecturer, and 52% for lecturer II. The MEPI program established and funded Staff Development Fellowships (SDFs) for graduate students training in areas of current teaching shortages who are committed to become faculty at UNZA or the new medical schools. These Staff Development Fellows are obligated to join faculty following their graduation for a minimum period equal to the duration of their graduate program. To this effect a total of 44 SDFs were supported by MEPI. SDF distribution by program is listed in Table 2. It was reported that this was the highest number of SDFs that had been recruited in the school from inception, a situation which was believed would help strengthen faculty and subsequently the quality of training (Table 2).

**Table 2:** Number of New SDFs

Degree Program	Study Program	Start Date	Number of new SDFs
**MSc**	Physiology	2012	6
	Biochemistry	2012	4
	Clinical Pharmacy	2012	2
	Anatomy	2012	5
	Haematology	2011	3
	Chemical Pathology	2011	6
	Medical Microbiology	2011	3
	Physiotherapy	2012	6
	Clinical Pathology	2012	6
**Master of Medicine**	Anaesthesia	2011	0
	Pathology	2011	0
	Psychiatry	2010	3
	Ophthalmology	2011	0
	Infectious Disease	2012	0
			**44**

**Development of new collaborations:** MEPI further contributed towards the expansion of the capacity of health worker education through facilitating the development of additional collaborations with the recently introduced schools of medicine in order to support the SDF program in the other schools. From 1972 to 2008, UNZA School of Medicine was the only medical school in Zambia. Since then, three new medical schools have been independently established in the country, namely the Copperbelt University School of Medicine which is a public institution and two private medical schools, Lusaka Apex Medical University and Cavendish University Zambia. While expanding the national capacity for training medical students, the introduction of these new schools also exacerbated the shortage of medical faculty, particularly in the basic sciences. To mitigate this shortage, UNZA SOM integrated and supported these new schools into the MEPI partnership locally. More importantly was the provision of support to the SDF program which aimed at sustaining faculty recruitment and retention for all the medical schools. It was stated that the Ministries of Health and Education supported these partnerships.

**Improving the quality of medical training:** Apart from improving the institutional capacity to train more health care workers, the MEPI program also facilitated processes for strengthening the quality of the training for health workers. This has been done through advancing technology in the medical laboratory and medical education sector.

**Integration of technology for medical education:** Data analysis indicated that following the implementation of the MEPI program, the SOM had new technologies installed which facilitated improvement in training process. Below the new technology which was installed is discussed in detail.

***The eGranary digital library:*** The eGranary digital library is an offline repository of internet content initially developed by the University of Iowa for low-resource settings with inadequate internet access. It provides learning resources that includes video, audio, books, journals, and web sites. With help from the University of Alabama, the Medical schools in Zambia installed eGranary units at all their campuses. To improve easy access to relevant materials by members of staff as well as students, it was reported that the library was transformed from a manual card cataloguing system to an electronic cataloguing system that is linked online to the main library. In addition, satellite libraries within the school's academic departments were established.

***Moodle course management software:*** The Moodle course management software has been installed where course materials developed by the faculty were supposed to be uploaded onto the platform. More than 24 staff had been trained on how to use it. It was reported that the platform had enhanced student access to research and training materials. Furthermore, this had also helped promote access and use of materials amongst faculty.

***REDCap:*** REDCap, the Research Electronic Data Capture system, is a user-friendly database system developed by the Vanderbilt University and licensed free of charge to participating institutions of higher learning (Citation: http://project-redcap.org). UNZA MEPI facilitated the installation of REDCap on servers at UNZA SOM, where it was being piloted for future wide-scale use by students, faculty, and research staff. This installation enhanced research activities by improving the scope of readily available data.

***Virtual microscopy:*** Another important innovation is the setting up of the Copperbelt University Medical School's virtual microscopy which is being used for histology and partially for histopathology. Virtual microscopyis a computer based method of training, in which optical microscopy images can be viewed as computer images. All manipulations, which can be done on a Microscopy can be done on the virtual microscopy images. The virtual microscopy had been used in the two undergraduate programs currently offered in the school, which are the Bachelors in Dental Surgery (BDS) and the Bachelor in Medicine and Surgery (MBChB). A total of 126 students have been taught Histology and Pathology using this program. Over the last 2 years in which the virtual microscopy has been used, the students had given virtual microscopy a favorable review. Documentary review further indicated that the students' performance in Histology was 10-20% better than their performance in Gross Anatomy and they attributed this performance partly to the new innovation.

***PowerLab and skills laboratory:*** Strengthening of the quality of training also involved the installation of the PowerLab Data Acquisition Systems in the Physiology and Pharmacology Laboratories at UNZA SOM. Five laboratory technicians were trained on its use in 2012. All students in undergraduate laboratory based courses at UNZA, LAMU and Cavendish begun utilizing the Powerlab. It was reported that the Powerlab was effective as it offered students the versatile and comprehensive data recording, display and analysis features for life science experiments in human Physiology, Pharmacology and other Biological courses as well as allowed quicker turnaround of experiments and shorter lab sessions. This enabled more lab sessions to be carried out in a single day than previously and further allowed the UNZA SOM physiology laboratory to be utilized by partner schools for their students. In addition, there were early successes in the management of the clinical skills simulation laboratory, where clinical skills were learned in a safe, standardized, and controlled environment.

***Improved processes for retaining faculty:*** The MEPI program has further contributed towards expansion of health care worker education in Zambia by putting in place approaches for improving retention of faculty. Compared to the situation before the MEPI program, it was reported that there was improvement in the following sectors following the implementation of the program: the academic environment, research support and offering more support for doctoral and postdoctoral levels.

***Strengthening academic environment:*** Strategies for transforming the academic environment into a conducive place included leveraging other support mechanisms in the school such the Welcome Trust supported research Support Centre activities on professional development and grant management training to faculty and support staff. This included training in medical education courses on curricula development, monitoring and implementation as well as courses on pedagogical approaches in medical education. Faculty were also given course and program coordination roles. All of these helped in stimulating interest in faculty to stay and work harder.

***Research support:*** Another activity for facilitating retention of faculty was research support. This was done by setting up analytical support processes (clinics, workshops for scientific writing and selected didactic basic and advanced courses) as well as research administration trainings. The archival and documentation system for the Research Ethics Committee were also strengthened. Training reports indicated that staff appreciated the training as they noted that the trainings had motivated them to engage in research activities.

***Doctoral and post-doctoral training support:*** A doctoral training support was started in the 2012 targeting faculty in PhD training. Support for this largely comprised travel and data analysis related support. This support has been integrated with similar efforts aimed at developing training capacity based consortia such as the Southern African Consortium for Research Excellence which focuses on doctoral and post-doctoral training, and is supported by the Wellcome Trust. It was reported that this integration process was vital as it may trigger improvement in training processes, commitment and retention of faculty.

**Implementation process limitations. Increased workload among faculty members:** Although the MEPI program triggered some positive developments, interviews showed that in the short term, implementation of the new technology and increased student enrollment amplified workload among faculty members. Due to increased workload, a lot of courses were not fully developed for uploading on to Moodle. In addition, standard operating procedures and feedback mechanisms for Moodle had not been developed.

## Discussion

In this paper, we have described the processes for strengthening and expanding the national capacity, quality for training health care workers, and retaining faculty through implementing the MEPI program in Zambia. Strategies that facilitated such improvements included the installation of new technology and the enhanced academic and research environment as well as leveraging of existing and new partnerships with other universities. Improved capacity by learning institutions to enroll more students is important as this addresses one of the major limitations to the contribution of most African medical schools towards addressing the HRH crisis which is the limited capacity by Universities to enroll a reasonable number of students [16–18]. Studies in other LMICs have shown that strategies, such as those used by the SOM, have the potential of contributing towards addressing HRH shortages [5, 12, 19–21]. To minimize the challenges that may arise because of increase in student enrollment, the MEPI program supported the SDF approach which could provide a sustainable measure to strengthening faculty. The approach has the potential of enabling a balance between the increased number of students enrolled in the medical schools and maintaining the quality of the training [22–24]. Another strategy for ensuring that increased student enroll does not affect quality of education was the installation of new technology. Installation of technology was made possible through leveraging existing collaborations. Use of technology in teaching is a critical component for improving the quality of training as it helps ease the teaching burden that accompanies rapid scale up of student intake and also improve access to information for research activities [22].

Meanwhile for technology to be effective, there is need to improve ICT infrastructure [25]. In addition, it is important to train faculty in the new technology if such technology is to be effectively adopted in medical schools in Africa. Training facilities adoption as it triggers compatibility of the new innovation with the contextual knowledge levels [26]. The roles that collaborations and local support play in improving the academic work environment are other important aspect of the MEPI program. Apart from strengthening existing collaborations, new ones have been developed at the local level following the implementation of the program. Such local partnerships are vital as they facilitate strengthening of synergies, collaborative priority setting and better utilization of limited resources [27]. The local support of research activities and postdoctoral fellowship provide a social structure for enabling faculty to expand the skill base and subsequently promote work motivation. This structure is relevant as it provides opportunities for faculty to advance their agency which is the capacity to explore new undertakings [15, 28]. In addition, a supportive academic environment can also help reduce attrition rates as it can stimulate increased interest in the job [29] . The MEPI strategy could arguably be the beacon that could help the medical universities provide new hope for increasing the number of HRH and possibly contribute towards improved health outcomes in Zambia [16, 30]. Integration of the program within the local management systems could provide sustainability of program processes and health systems [31–33]. The MEPI program could be a potential response to common global challenges surrounding health worker shortages such as skill mix imbalance, mal-distribution, negative work environment, a weak knowledge base and lack of investment [34].

**Study limitations and strengths:** Not being able to interview the students who are currently using the new technology that has been put in place by the MEPI program was one of the study limitations. This denied the study additional perspectives on the compatibility of the technology. To mitigate this limitation, the study included data from student course assessment reports. Although generalizability was not the intention, the rich description of processes for strengthening the capacity for health worker education and retaining faculty as well as our multiple methods of data collection helped in developing an account that we believe provides a valuable contribution to the knowledge base on this subject that can provide useful lessons in other LMICs.

## Conclusion

The implementation of MEPI program has resulted into processes and systems for improving the capacity and quality of training processes in medical schools as well as strengthened systems for retaining faculty. The capacity to enroll more students has been made possible through the development of new programs and differentiating old Master in Public Health into specialized curriculum. Whereas the quality of training was enhanced through integration of technology in training processes and library. This new technology enhanced access to relevant data for training and research as well as improved the features of science experiments. Improving the academic work environment, increased support towards research and staff development program has the potential of improving retention of faculty as these stimulated work motivation and interest in research among faculty.

### What is known about this topic

- The situation of human resource for health (HRH) crisis in Zambia and other low and middle income countries;
- Causes of HRH crisis;
- Effects of HRH crisis on health service delivery.

### What this study adds

- The role of local and international partnerships in improving the quality of medical education in a low-income setting;
- Experience of integrating new technology in medical education in a low-income setting;
- Relevance of integrating new technology in medical education in a low-income setting.

## Competing interests

The authors declare no competing interest.
